# Advanced fabrication of biosensor on detection of Glypican-1 using S-Acetylmercaptosuccinic anhydride (SAMSA) modification of antibody

**DOI:** 10.1038/s41598-018-31994-2

**Published:** 2018-09-10

**Authors:** Yifan Dai, Kevin Abbasi, Michael DePietro, Samantha Butler, Chung Chiun Liu

**Affiliations:** 1 Department of Chemical and Biomolecular Engineering, Case Western Reserve University, 10900 Euclid Ave, Cleveland, OH 44106 USA; 2 Electronics Design Center, Case Western Reserve University, 10900 Euclid Ave, Cleveland, OH 44106 USA; 3 Swagelok Center for Surface Analysis of Materials (SCSAM), Case Western Reserve University, 10900 Euclid Ave, Cleveland, OH 44106 USA

## Abstract

Glypican-1 (GPC-1) has been recognized as biomarker of pancreatic cancer. Quantification of GPC-1 level is also pivotal to breast cancer and prostate cancer’s patients. We hereby report the first biosensor for GPC-1 detection. Instead of using crosslinking technique and surface immobilization of antibody, we applied a novel method for biosensor fabrication, using S-Acetylmercaptosuccinic anhydride (SAMSA) to modify the Anti-GPC-1 producing a thiol-linked Anti-GPC-1. The thiol-linked Anti-GPC-1 was then directly formed a single-layer antibody layer on the gold biosensor, minimizing the biosensor preparation steps significantly. Time of Flight Secondary Ions Mass Spectroscopy (TOF-SIMS) characterization verified the thiol-linked antibody layer and demonstrated a unique perspective for surface protein characterization. Differential pulse voltammetry (DPV) was applied to quantify GPC-1 antigen in undiluted human serum with a concentration range of 5,000 pg/µL to 100 pg/µL. The performance of this newly designed biosensor was also compared with modified self-assembled monolayer system fabricated biosensor, demonstrating the high-sensitivity and high-reproducibility of the SAMSA modified antibody based biosensor. This simple fabrication method can also expand to detection of other biomolecules. The simplified operation process shows great potential in clinical application development.

## Introduction

Glypican-1 (GPC-1) was identified in June 2015 as a biomarker for early detection of pancreatic cancer, it was reported that the detection of GPC-1 was 100% correct for 250 patients^1^. This report generated much interest in the detection of this biomarker of early stage of pancreatic cancer. Other studies also suggested that GPC-1 was a biomarker for pancreatic cancer^2,3^. Scientifically, there were reservations about whether GPC-1 could be a real early biomarker of pancreatic cancer. The rational for this reservation was based on^1^ the 70–89% of the patients studied were at the advanced stage –IIb, III and IV stages of pancreatic cancer and^2^ only five intraductal papillary mucinous neoplasm (IPMN), a precursor of pancreatic cancer was included in the study^4,5^. IPMN lesions do not always evolve in malignant tumors^2,6^. While the scientific and clinical discussion of the nature of the GPC-1 related to the stage of the pancreatic cancer continued, nevertheless, many studies showed that GPC-1 was crucial for efficient cancer cell growth, metastasis and in the pathogenesis of diseases. This included Alzheimer’s disease^7,8^, prion disease^9–11^ and others^5,8,12^. Therefore, the detection of GPC-1 will be meaningful in the assessment and the observation of the progression of cancer and neuro-degenerative disorders in general. Furthermore, glypican-1 has been identified in human blood^1^, producing a promising potential for rapid detection of GPC-1 for clinical assessment.

Ultracentrifugation was done at high speed and overnight to isolate exosomes from serum sample and then GPC-1 was detected using electron microscope^1,5^. Other studies on the detection of GPC-1 used tissue samples^13^. Confocal immunofluorescence microscopy was also employed in some of the studies of GPC-1^14^. These evaluations of GPC-1 level provided useful and accurate results. However, the techniques used were elaborate and required expensive equipment and skilled operator. Therefore, it is desirable to design a simple detection method of GPC-1 providing a useful tool for the assessment of the level of GPC-1 and its effect in cancers and neuro-degenerative disorders. Specifically, a single-use, disposable *in vitro* biosensor was the objective of this development endeavor. This GPC-1 biosensor was manufactured by using a cost-effective, industrial scale fabrication method to produce a single-use, disposable device that was relatively inexpensive^15^. This biosensor is based on thin gold-film working and counter electrode with Ag/AgCl based reference electrode. The performance of the stability and reproducibility of this biosensor had been demonstrated in our previous study^15^.

For biosensor fabrication, conventional SAM monolayer systems have demonstrated an excellent platform for immobilization of biomolecules^15–18^. However, the complex operating procedures and the tedious preparation steps for biosensor fabrication promote the development of a simpler method for biosensor fabrication. Bioconjugation chemistry has demonstrated an assuring method for biosensor fabrication, providing a shorter process for fabrication of antibody based biosensor^19^. In this study, we studied the performance of S-Acetylmercaptosuccinic anhydride (SAMSA) on biosensor fabrication. SAMSA was used to produce the external thiol linker to the Anti-GPC-1, which can then be directly linked to the gold electrode surface without any surface modifications. Short link length between gold surface and the antibody provides a better coverage and less possibility of pinhole formation for the biosensor surface^20,21^, which can significantly increase the sensitivity and resolution of the biosensor comparing with those of traditional SAM prepared biosensor. Also, the simplified surface configuration based on SAMSA modified antibody (only a single-layer of antibody) is a much more efficient layer for analyte absorption comparing with that of SAM system. The simplified surface layer not only prevents antigen non-specific binding, but also avoids the decrease of electrochemical signal caused by complex, multi-layer, fluctuated organic surface. We hereby present and discuss the difference of a well-defined SAM system^22^ (3-Mercaptopropionic acid (3-MPA) and dithiothreitol (DTT)) prepared biosensor and a SAMSA prepared biosensor on their performance on GPC-1 detection.

## Results and Discussions

### Characterization of SAMSA Conjugation Process

**Figure 1:**
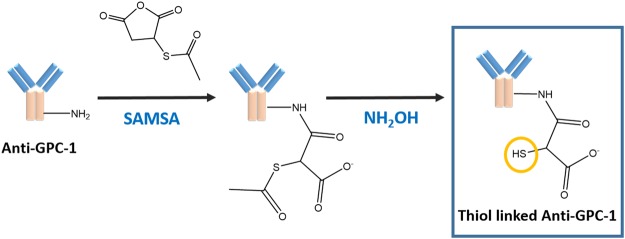
Reaction process of SAMSA and Anti-GPC-1 producing thiol-linked Anti-GPC-1.

For biosensor fabrication based on gold electrode, the most pivotal step is formation of the Au-S group for the introduction of antibody, enzyme or receptor onto the biosensor surface in order to detect the specific analyte. In this study, we demonstrated the use of S-Acetylmercaptosuccinic anhydride (SAMSA) to introduce the thiol group onto the anti-GPC-1 for detection of GPC-1 antigen. The thiol linked antibody further directly incubated onto the gold electrode, providing a single-step for biosensor fabrication (1 hr), which significantly shortened the biosensor fabrication period comparing with that of SAM method (3 days). The reaction process of SAMSA and anti-GPC-1 is demonstrated in Fig. 1. The whole reaction process of conjugation of antibody is around two and half hours. The conjugated anti-GPC-1 solution was further stored at −20 °C for future use.

**Figure 2:**
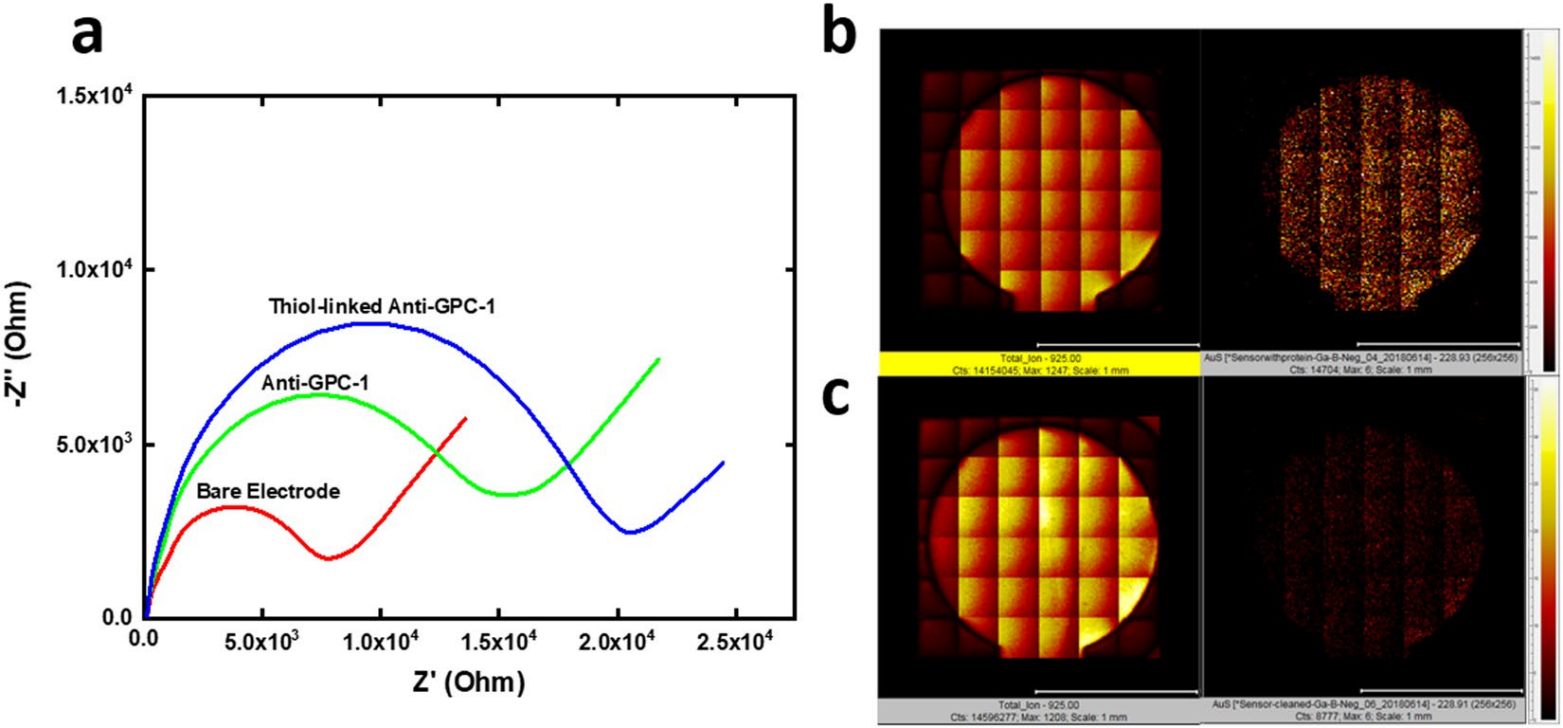
(**a**) Nyquist plot for impedance characterization of bare electrode, Anti-GPC-1 incubated electrode, and thiol-linked Anti-GPC-1 incubated electrode.

We characterized the reactivity of the external thiol linker on the Anti-GPC-1 by analyzing the electrochemical impedance difference before and after the incubation of thiol linked Anti-GPC-1. We also compared thiol-linked Anti-GPC-1 with non-modified anti-GPC-1 on their affinity to gold electrode surface. As shown in Fig. 2a, Nyquist plot represents the impedance difference among bare electrode, Anti-GPC-1 incubated electrode, and thiol-linked Anti-GPC-1 incubated electrode. Experimentally, 7.5 µg/mL of thiol-linked Anti-GPC-1 and the same concentration of unmodified Anti-GPC-1 solution were both incubated onto the electrode for 1 h at room temperature. After incubation, the biosensors were cleaned by deionized water and dried by nitrogen gas prior to the electrochemical impedance spectroscopy (EIS) test. The EIS test was conducted with the presence of redox coupling [Fe(CN)6]^3−/4−^. As shown in Fig. 2a, unmodified Anti-GPC-1 protein solution showed some gold-affinity, because amino acids such as cysteine, penicillamine, and glutathione, are thiol contained molecules in the protein. Therefore, they can provide natural affinity to gold^23^. However, thiol-linked Anti-GPC-1 shows a much bigger impedance value comparing with that of unmodified Anti-GPC-1, indicating the validity of the Au-S reaction.

Surface characterization of GPC-1 biosensor was also conducted by Time of Flight Secondary Ion Mass Spectroscopy (TOF-SIMS). Elements maps of total ions and Au-S group are shown in Fig. 2b,c for thiol-linked Anti-GPC-1 incubated biosensor and unmodified Anti-GPC-1 incubated biosensor. The elements map of Au-S in Fig. 2b showed a homogeneous distribution of Au-S group, indicating an excellent distribution of the Anti-GPC-1 antibody on the working electrode surface. However, the elements map of Au-S in Fig. 2c demonstrated 60% less counts of Au-S group comparing with that of Fig. 2b, confirming that the effectiveness of the SAMSA reaction process on providing the external thiol-linker onto the Anti-GPC-1 and its reactivity with gold electrode surface.

**Figure 3:**
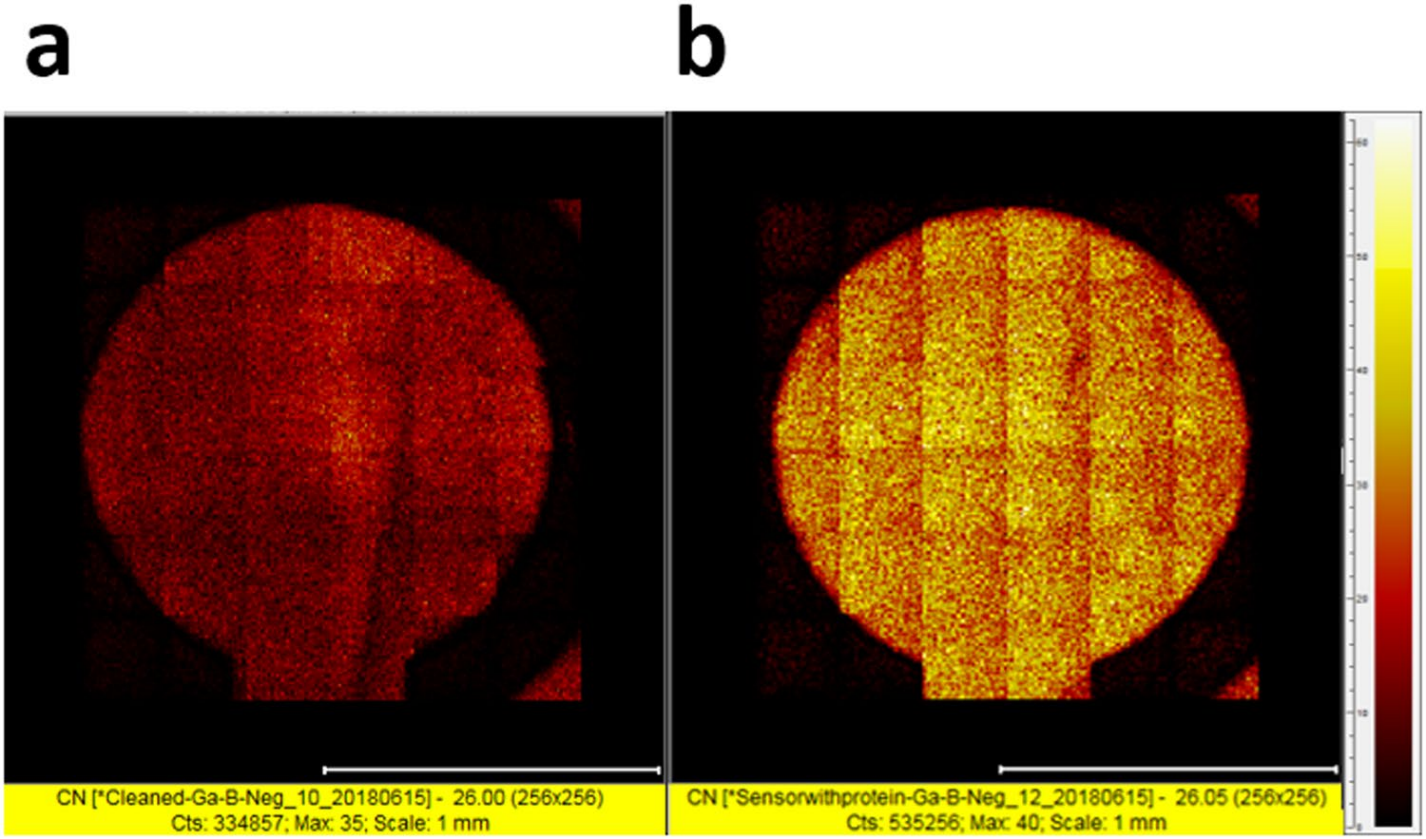
TOF-SIMS analysis of C-N bond based on two biosensors, (**a**) unmodified Anti-GPC-1 incubated electrode surface; (**b**) thiol-linked Anti-GPC-1 incubated electrode surface.

Another interesting finding from TOF-SIMS was that the counts of carbon-nitrogen (C-N) bonds in thiol-linked Anti-GPC-1 incubated biosensor increased significantly for about 1.6 times comparing with that of unmodified Anti-GPC-1 incubated biosensor as shown in Fig. 3. We already demonstrated the thiol-linked protein displayed higher affinity on the gold electrode. Carbon-nitrogen bond, as one of the most distributed bonds in the amino acids, is an eligible representative of protein. Therefore, the characterization of C-N bond using TOF-SIMS provides a new perspective for surface protein analysis and a potential for surface protein quantification. Also, these results combined proved the effectiveness of fabrication of biosensor in 1 hr.

### Electrochemical Characterization of the GPC-1 Biosensor

**Figure 4:**
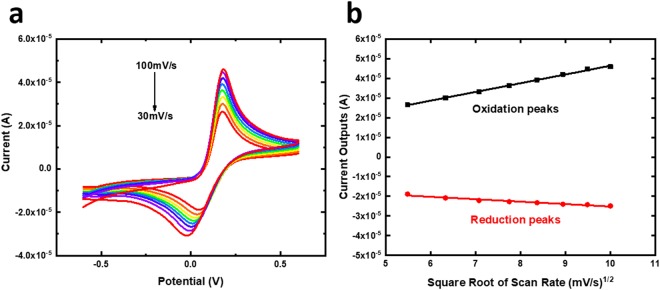
(**a**)CV characterization of GPC-1 biosensor based on scan rates of 30 mV/s–100 mV/s. (**b**) Linear calibration curves of the oxidation peaks and the reduction peaks against the square root of scan rate based on CV characterization of GPC-1 biosensor.

Cyclic voltammetry (CV) characterization of sensing device based on Randle-Sevcik Equation is a well-validated method to prove the stability and reproducibility of SAMSA modified Anti-GPC-1 produced biosensor^15,24^. Typically, a well-calibrated sensor under CV should embrace a linear relationship between the current peak outputs and the square root of scan rates at the condition with the same redox event, constant diffusion constant and identical temperature. As shown in Fig. 4a, the CV current outputs for both oxidation and reduction states at the presence of [Fe(CN])_6_]^3−/4−^ were examined at scan rates from 30–100 mV/s. Fig. 4b demonstrates the linear relationship between the square root of scan rates against the current outputs of both oxidation and reduction with an average R-square value of 0.99785, indicating an excellent reproducibility and stability of the GPC-1 biosensor.

### Differential pulse voltammetry quantification of GPC-1 antigen

**Figure 5:**
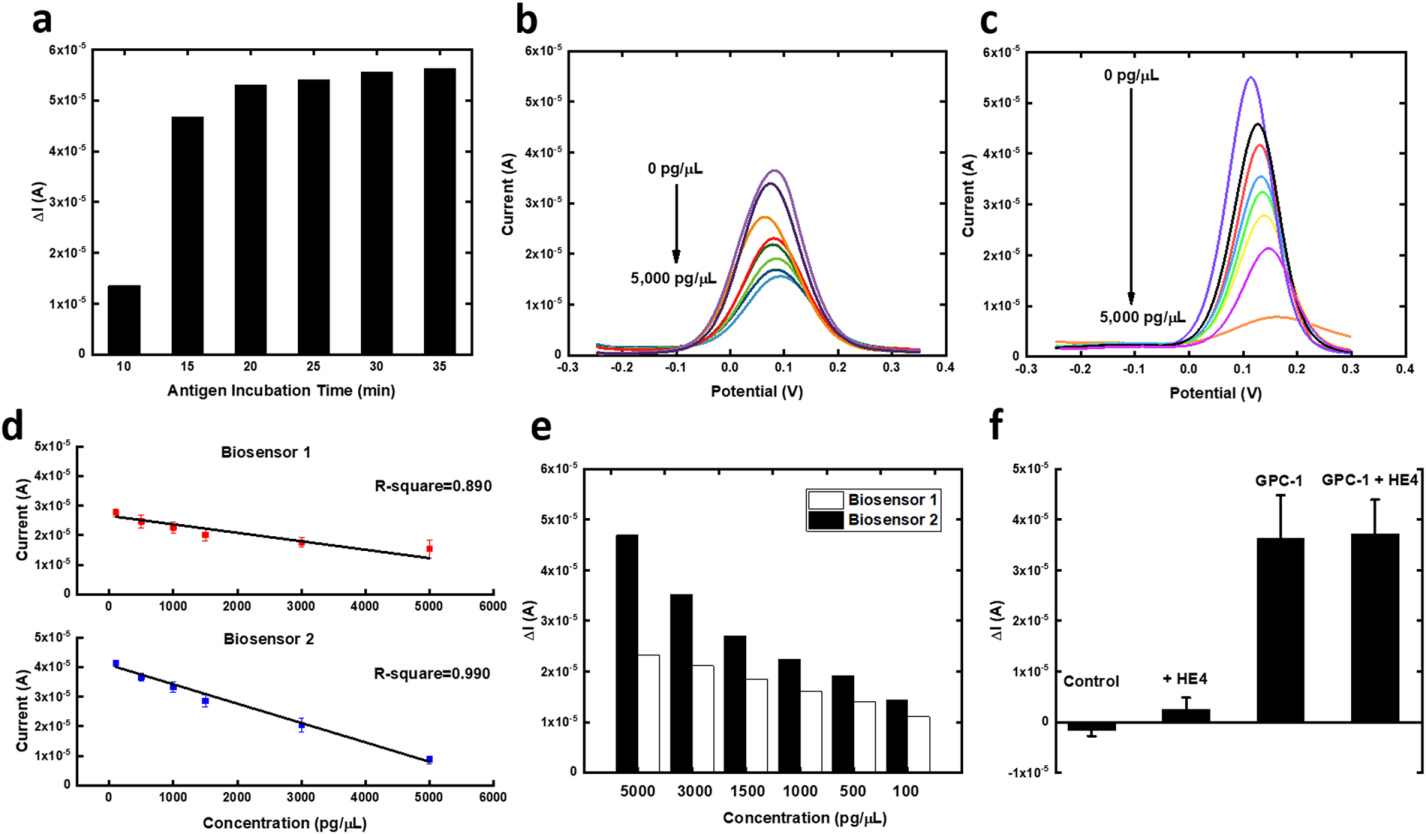
(**a**) Investigation of different antigen incubation time on GPC-1 biosensor. (**b**) Modified self-assembled monolayer system (3-MPA & DTT) based GPC-1 biosensor (Biosensor 1) on DPV measurements of multiple concentrations of GPC-1 antigen in undiluted human serum. (**c**) SAMSA modified antibody based GPC-1 biosensor (Biosensor 2) on DPV measurements of multiple concentrations of GPC-1 antigen in undiluted human serum. (**d**) The calibration curve based on Biosensor 1 and Biosensor 2. (**e**) Exhibition of change of current response at different concentrations of GPC-1 antigens based on two different biosensors. (**f**) Interference study based on HE4 antigen.

Differential pulse voltammetry (DPV) is widely recognized as a high-sensitivity electrochemical transduction mechanism for electrochemical sensing applications^15,25–27^. The performance of the GPC-1 biosensor fabricated by SAMSA was compared with the GPC-1 biosensor fabricated through SAM system, because SAM has been proved to be an effective and popular technique for biosensor development. The GPC-1 biosensor fabricated through modified SAM system (Biosensor 1) and the GPC-1 biosensor fabricated by SAMSA modified antibody (Biosensor 2) were both prepared using the same concentration of antibody (7.5 µg/mL) and further incubated at different concentrations of GPC-1 antigen in undiluted human serum for performance comparison using DPV measurements. The optimized incubation time was firstly evaluated by incubating the 5,000 pg/µL GPC-1 antigen sample for different time periods. As shown in Fig. 5a, the change of current was unchanged during 20–30 min, so 30 min was selected as the optimized antigen incubation time.

Different GPC-1 antigen concentrations ranging from 5,000 pg/µL to 100 pg/µL (in undiluted human serum) were then tested by DPV with the presence of [Fe(CN)_6_]^3−/4−^. [Fe(CN)_6_]^3−/4−^ is a well-defined redox system, which has been widely used in protein, nucleic acid quantifications^15,28,29^. Current outputs showed reverse proportional to the concentration gradients, because higher the concentration of GPC-1 antigen would decrease the conductivity on the biosensor surface due to increasing surface resistivity. Figure 5b shows the DPV measurement of GPC-1 antigen through Biosensor 1 and Fig. 5c shows the DPV measurement of GPC-1 antigen through Biosensor 2. Comparing between these two types of biosensors, Biosensor 2 demonstrated a higher sensitivity under the same concentration range. The higher current outputs from the SAMSA fabricated GPC-1 biosensor indicated the lower surface impedance on the biosensor surface, which was brought by the simplified biosensor surface produced by SAMSA conjugated antibody. Comparing with the Biosensor 1, Biosensor 2 only possessed a single layer of antibody, a much simplified surface structure comparing with multiple immobilization layers in the SAM system. This simplified surface also ensured the high-sensitivity of the SAMSA modified antibody fabricated GPC-1 biosensor. The linear calibration curves of these two types of GPC-1 biosensors are shown in Fig. 5d. Biosensor 2 not only demonstrated a higher R-square value of 0.990 comparing with that of Biosensor 1, but also showed a lower RSC value of 3.36%, indicating an excellent reproducibility was achieved by the SAMSA modified antibody fabricated GPC-1 biosensor.

Another finding was that the range of current change for the same concentration range was different based on these two methods fabricated biosensor as shown in Fig. 5e. Biosensor 2 showed a higher range of change of current for the same concentration range of GPC-1 antigen because of the simplified surface. The uniformity of the SAM system is apparently not comparable with the single-layer of antibody on the biosensor. The inhomogeneous SAM structures on the biosensor surface may hinder the antigen-antibody reaction, resulting a low absorption rate of antigen onto the biosensor surface comparing with that of Biosensor 2. Therefore, the range of change of current for Biosensor 1 was lower than that of Biosensor 2, indicating a higher resolution/recognition ability was achieved by the SAMSA modified antibody fabricated GPC-1 biosensor. Thus, Biosensor 2 was selected for further interference study.

### Interference study of the GPC-1 biosensor

In order to prove the selectivity of the novel fabrication method produced biosensor (Biosensor 2), HE4 antigen was applied for interference test. 5,000 pg/µL of HE4 antigen was incubated onto the GPC-1 biosensor for 30 min. As shown in Fig. 5f, the DPV output based on HE4 antigen showed the same current response as the zero concentration of GPC-1 antigen, indicating the HE4 antigen showed no absorption onto the anti-GPC-1 layer. We further mixed the HE4 antigen with GPC-1 antigen to evaluate any influence of HE4 antigen on GPC-1 antigen- antibody affinity. The change of current based on mixed HE4 antigen and GPC-1 antigen and solo GPC-1 antigen was also very similar. Both tests demonstrated an excellent selectivity based on the GPC-1 biosensor.

Taken together, the application of acetylmercaptosuccinic anhydride (SAMSA) on biosensor fabrication provides a unique perspective for the development of rapid, simple, effective biosensing system for detection of biomarkers. The use of SAMSA modified antibody significantly minimized the fabrication process of antibody based biosensor. The simplified electrode surface (with only a single-layer of antibody) promises a high-sensitivity sensing platform, which can be a versatile application method for detection of various biomarkers.

## Materials and Experimentals

### Regents and Apparatus

Antibody GPC-1 produced from rabbit (Cat. # HPA030571) and antigen of GPC-1 (Cat. # APREST 78918) were obtained from Sigma Aldrich (St. Louis, MO). Phosphate-buffered saline (PBS) 1.0 M (pH 7.4), S-Acetylmercaptosuccinic anhydride(SAMSA), human serum, 3-Mercaptopropionic acid (MPA), dithiothreitol (DTT), N-(3 dimethylaminopropyl)-N′-ethylcarbodiimide hydrochloride (EDC) and N–hydroxysuccinimide (NHS) were also purchased from Sigma-Aldrich (St. Louis, MO). Potassium hydroxide pellets, concentrated H_2_SO_4_ (95.0 to 98.0 w/w %) and concentrated HNO_3_ (70% w/w %) were received from Fisher Scientific (Pittsburgh, PA.). Recombinant human HE4 protein (Cat. # ab184603) was obtained from Abcam (Cambridge, MA). All chemicals were used without further purification. A CHI 660 C (CH Instrument, Inc., Austin, TX) Electrochemical Workstation was used for DPV and EIS investigations. All the experiments were conducted at room temperature.

### S-Acetylmercaptosuccinic anhydride (SAMSA) modification of anti-GPC-1

SAMSA was firstly dissolved by dimethyl sulfur oxide (DMSO) at a concentration of 0.5 mg/mL. 5 µL of the stock SAMSA solution was added into 50 µL of 1 mg/mL GPC-1 antibody solution in 0.1 M PBS buffer containing 0.15 M NaCl. The reaction ratio between SAMSA and antibody was based on 20:1 molar ratio^30^. The mixed solution was reacted at room temperature for 30 min. The solution was then transferred into Amicon ultra-0.5 10 k filter and centrifuged at 12,000 rpm for 15 min at 5 °C removing excess reagent and any by-products. This filtered antibody solution was then applied to further deacetylation process. 0.5 M hydroxylamine hydrochloride solution in 0.1 M PBS containing 25 mM EDTA was applied for deacetylation process. 10 µL of the prepared hydroxylamine hydrochloride solution was added into 50 µL of filtered antibody solution. The volume of the filtered solution was altered by 0.1 M PBS buffer containing 0.15 M NaCl and 10 mM EDTA if necessary. The deacetylation reaction was taken at room temperature for 2 h. After the reaction, sulfhydryl-modified antibody solution was diluted to 500 µL and filtered again using Amicon ultra-0.5 10 k tube by centrifuging at 12,000 rpm for 15 min at 5 °C removing excess reagent and any by-products. The filtered solution was the final thiol-linked antibody solution. The concentration of the thiol-linked antibody solution was then determined by the absorptivity at 280 nm by ultraviolet light.

### Fabrication of GPC-1 biosensor

Prior to immobilization of the thiol linked antibody, 10–12 biosensors were firstly cleaned based an established protocol using KOH, H_2_SO_4_ and HNO_3_ solutions^15^. This step intended to enhance the reproducibility and sensitivity of the biosensor, which were also proved in our previous study^15^. After cleaning, the SAMSA conjugated antibody solution was diluted to a concentration 7.5 µg/mL using 0.1 M PBS buffer containing 0.15 M NaCl and 10 mM EDTA. The diluted antibody solution was drop-casted onto the cleaned biosensors with 20 µL of antibody solution per biosensor and incubated for 1 hr at room temperature to form the Au-S bond. After incubation, the biosensors were cleaned by 0.1 M PBS solution and dried by nitrogen gas flow. The GPC-1 biosensors were then stored at 4 °C and ready for further usage.

### Fabrication of GPC-1 biosensor based on modified SAM monolayer system

3-MPA and DTT were applied simultaneously. Typically, 1 mM MPA and 1 mM DTT were mixed in a 15 mL pure ethanol solution and a batch of 10 chemically pretreated biosensors were then immersed in the solution for 24 hours. The biosensors were then rinsed with DI water. The biosensors were then functionalized by incubating in 0.1 M PBS (pH = 7.4) containing 0.25M N-(3-dimethylaminopropyl)-N′-ethylcarbodiimide hydrochloride (EDC) and 0.05 M N–hydroxysuccinimide (NHS) for 5 hr at room temperature. The biosensors were then rinsed with DI water and dried by nitrogen gas. Then 16 µL of antibody of GPC-1 solution in a carbonate-bicarbonate buffer (pH = 9.6) with concentration at 7.5 µg/mL was placed onto the gold electrode surface and was incubated overnight at 4 °C. Basic buffer was used for incubating of GPC-1 antibody because basic solution condition maintained the amine group on the antibody as nucleophile, resulting in better binding with the ester group formed on the electrode surface. The GPC-1 antibody immobilized biosensors were then rinsed with 0.1 M PBS and immersed in 1 mM BSA solution for 30 min at room temperature, covering any unbounded ester sites produced by the MPA/EDC reaction. The biosensors were then rinsed with 0.1 M PBS, dried by nitrogen gas, and stored at 4 °C and ready for use. These preparation steps of the GPC-1 biosensors were accomplished prior to actual measurement of the GPC-1 antigen concentration in the test liquid medium.

### Electrochemical impedance spectroscopy (EIS)

Electrochemical impedance spectroscopy (EIS) was applied with a frequency range of 0.1–10,000 Hz at an amplitude of 0.05 V. Prior to EIS test, 20 µL of the 5 mM of redox coupling solution [Fe(CN)_6_]^3−/4−^ was dropped onto the biosensor surface.

### Time of Flight Secondary Ion Mass Spectroscopy (TOF-SIMS)

TOF-SIMS was conducted at negative polarity. Secondary-ion images were collected by distributing primary ion (Ga+) over the working electrode surface. Ga^+^ beam was accelerated to 30 kV with a pulse size of 7 ns. The acquisition rate was set at 8 kHz.

### Differential pulse voltammetry profiling of different GPC-1 antigen concentrations

Differential pulse voltammetry (DPV) was applied as electrochemical transduction mechanism. Prior to DPV test, the GPC-1 biosensor arrays were incubated with antigen for 30 min. After incubation of GPC-1 antigen, the biosensor arrays were immersed in 0.1 M PBS for 1 min to remove unlinked GPC-1 antigen and further dried by nitrogen gas. −0.25 V to +0.3 V was the voltage scan range of DPV with the increment of 0.004 V, pulse width of 0.05 s, amplitude of 0.05 V, sampling width of 0.0167 s and pulse period of 0.2 s. 20 µL of the 5 mM of redox coupling solution [Fe(CN)_6_]^3−/4−^ was dropped onto the biosensor surface previous to the DPV test.
